# Conventional risk prediction models fail to accurately predict mortality risk among patients with coronavirus disease 2019 in intensive care units: a difficult time to assess clinical severity and quality of care

**DOI:** 10.1186/s40560-021-00557-5

**Published:** 2021-06-01

**Authors:** Hideki Endo, Hiroyuki Ohbe, Junji Kumasawa, Shigehiko Uchino, Satoru Hashimoto, Yoshitaka Aoki, Takehiko Asaga, Eiji Hashiba, Junji Hatakeyama, Katsura Hayakawa, Nao Ichihara, Hiromasa Irie, Tatsuya Kawasaki, Hiroshi Kurosawa, Tomoyuki Nakamura, Hiroshi Okamoto, Hidenobu Shigemitsu, Shunsuke Takaki, Kohei Takimoto, Masatoshi Uchida, Ryo Uchimido, Hiroaki Miyata

**Affiliations:** 1 Department of Healthcare Quality Assessment, Graduate School of Medicine, The University of Tokyo, 7-3-1 Hongo, Bunkyo-ku, Tokyo, 113-8655 Japan; 2 Department of Health Policy and Management, School of Medicine, Keio University, 35 Shinanomachi, Shinjuku-ku, Tokyo, 160-8582 Japan; 3 Department of Clinical Epidemiology and Health Economics, School of Public Health, The University of Tokyo, 7-3-1 Hongo, Bunkyo-ku, Tokyo, 113-0033 Japan; 4 Department of Critical Care Medicine, Sakai City Medical Center, 1-1-1 Ebaraji-cho, Nishi-ku, Sakai, Osaka, 593-8304 Japan; 5 Intensive Care Unit, The Jikei University School of Medicine, 3-19-18 Nishi-Shinbashi, Minato-ku, Tokyo, 105-8471 Japan; 6 Department of Anesthesiology and Intensive Care Medicine, Kyoto Prefectural University of Medicine, 465 Kajii-cho, Kawaramachi-Hirokoji, Kamigyo-ku, Kyoto, 602-8566 Japan; 7 Department of Anesthesiology and Intensive Care Medicine, Hamamatsu University School of Medicine, 1-20-1 Handayama, Higashi-ku, Hamamatsu, Shizuoka, 431-3192 Japan; 8 Intensive Care Unit, Kagawa University Hospital, 1750-1 Ikenobe, Miki-cho, Kita-gun, Kagawa 761-0793 Japan; 9 Division of Intensive Care, Hirosaki University Hospital, 53 Honcho, Hirosaki, Aomori, 036-8203 Japan; 10 Department of Emergency and Critical Care Medicine, National Hospital Organization Tokyo Medical Center, 2-5-1, Higashigaoka, Meguro-ku, Tokyo, 152-8902 Japan; 11 Department of Emergency and Critical Care Medicine, Saitama Red Cross Hospital, 1-5 Shintoshin, Chuo-ku, Saitama, 330-8553 Japan; 12 Department of Anesthesiology, Kurashiki Central Hospital, 1-1-1 Miwa, Kurashiki, Okayama, 710-8602 Japan; 13 Department of Pediatric Critical Care, Shizuoka Children’s Hospital, 860 Urushiyama, Aoi-ku, Shizuoka, Shizuoka 420-8660 Japan; 14 Department of Pediatric Critical Care Medicine, Hyogo Prefectural Kobe Children’s Hospital, 1-6-7 Minatojima Minamimachi, Chuo-ku, Kobe, Hyogo 650-0047 Japan; 15 Department of Anesthesiology and Critical Care Medicine, Fujita Health University School of Medicine, 1-98 Dengakugakubo, Kutsukake-cho, Toyoake, Aichi 470-1192 Japan; 16 Department of Critical Care Medicine, St. Luke’s International Hospital, 9-1 Akashi-cho, Chuo-ku, Tokyo, 104-8560 Japan; 17 Department of Intensive Care Medicine, Graduate School of Medicine, Tokyo Medical and Dental University, 1-5-45 Yushima, Bunkyo-ku, Tokyo, 113-8519 Japan; 18 Department of Anesthesiology and Critical Care Medicine, Yokohama City University, 3-9 Fukuura, Kanazawa-ku, Yokohama, Kanagawa 236-0004 Japan; 19 Department of Intensive Care Medicine, Kameda Medical Center, 929 Higashi-cho, Kamogawa, Chiba, 296-8602 Japan; 20 Department of Emergency and Critical Care Medicine, Dokkyo Medical University, 880 Kitakobayashi, Mibu-machi, Shimotsuga-gun, Tochigi, 321-0293 Japan

**Keywords:** Coronavirus disease 2019, Risk of death, Intensive care unit, Risk prediction model, Quality improvement

## Abstract

Since the start of the coronavirus disease 2019 (COVID-19) pandemic, it has remained unknown whether conventional risk prediction tools used in intensive care units are applicable to patients with COVID-19. Therefore, we assessed the performance of established risk prediction models using the Japanese Intensive Care database. Discrimination and calibration of the models were poor. Revised risk prediction models are needed to assess the clinical severity of COVID-19 patients and monitor healthcare quality in ICUs overwhelmed by patients with COVID-19.

Dear Editor,

Since the start of the coronavirus disease 2019 (COVID-19) pandemic, intensive care units (ICUs) worldwide have struggled to treat affected patients who require a completely different approach to treatment than other patients [1]. Although many severe cases are admitted to ICUs, it is unknown whether the conventional risk scoring systems that were developed for ICU patients can be applied to patients with COVID-19. With unknown predictive performance, healthcare professionals have faced difficulties in assessing the clinical severity of patients with COVID-19 and monitoring the quality of care in ICUs. New risk prediction models for COVID-19 patients have been developed [2], but most of these were not developed specifically for ICU patients, and it is unknown whether they perform as well in clinical practice as they did in the model development studies. It is also likely that overwhelmed ICUs lack the time to derive and validate novel risk scores. In such circumstances, ICUs must use conventional scoring systems, such as the Acute Physiology and Chronic Health Evaluation (APACHE) and Simplified Acute Physiology Score (SAPS). Several recent studies have used APACHE and SAPS to provide information on the clinical severity of COVID-19 [3–5]. However, very few reports have examined their validity of applying them to patients with COVID-19. One letter from the UK reported that APACHE II underestimated the risk of death, concluding that the risk scoring systems that were widely used before the pandemic were inappropriate for evaluating the clinical severity of COVID-19 [6]. In Japan, a research group recently developed the Japan Risk of Death (JROD), a prediction model that recalibrated the APACHE III-j model [7]. However, this model may show limited validity in patients with COVID-19 because it was developed using the data collected before the pandemic and it was designed for general use in ICUs. Therefore, we investigated whether conventional risk prediction models, such as APACHE II, SAPS II, APACHE III-j, and JROD, can be applied to patients with COVID-19 and determined their predictive performance.

We obtained data for confirmed cases of COVID-19 admitted between January 2020 and February 2021 from the Japanese Intensive Care Patient Database (JIPAD) [8]. We used JROD to predict mortality in the same way as in the previous study [7], but with a development period of January 2019 to December 2019. This was then applied to predict mortality in the study cohort and defined as JROD_2019_ predicted mortality. The predictive performances of APACHE II, SAPS II, APACHE III-j, and JROD_2019_ were assessed using the area under the receiver operating characteristic curves, Brier scores, Hosmer–Lemeshow tests, calibration plots, and standardized mortality ratios.

A total of 444 patients admitted to 40 ICUs in Japan were extracted from the JIPAD for analysis. The clinical characteristics of patients are shown in Table 1. The model performance statistics are presented in Table 2 and Fig. 1. Death at hospital discharge was recorded in 69 patients (15.5%), which was less than half the mortality reported by Stephens et al., although the APACHE II scores were comparable [6]. Using JIPAD data, the APACHE II, SAPS II, and APACHE III-j models overestimated the risk of death, whereas JROD_2019_ underestimated the risk. The discrimination and calibration of APACHE III-j and JROD were poor compared with those reported in the JROD development study [7]. Although the results are dissimilar to a previous report [6] in terms of the direction of estimated risk (i.e., overestimation/underestimation), we make the same conclusion that the risk models used before the pandemic are not suitable for patients with COVID-19. Of note, even JROD_2019_, a model that was developed to improve the predictive ability of APACHE III-j, displayed suboptimal predictive performance. Owing to the poor predictive performance, it is difficult to incorporate the predicted mortality calculated using these risk models in quality assessment tools, such as funnel plots and exponentially weighted moving average charts, with high reliability. Consequently, it will be difficult to implement quality assessment and improvement in ICUs, particularly those where patients with COVID-19 occupy a high proportion of ICU beds. Calibration can be improved with simple update methods, like that done in the JROD study, but discrimination can only be improved by updating the coefficients of each predictor and/or adding other relevant predictors [9]. Thus, a revised risk prediction model designed specifically for COVID-19 patients together with logistical support for its implementation in ICUs are urgently needed.

**Table 1:** Clinical characteristics

Characteristic	Value
**Number of patients**	444
**Baseline characteristics**	
Age, years, median [IQR]	68 [58, 74]
Male (%)	342 (77.0)
Body mass index, kg/m^2^, median [IQR]	25 [22, 28]
Days from hospital admission to ICU admission, median [IQR]	0 [0, 1]
Admission source (%)	
Emergency room	141 (31.8)
Transfer from another hospital	159 (35.8)
Ward	129 (29.1)
Other	15 (3.4)
APACHE II score, median [IQR]	16 [13, 21]
APACHE II predicted mortality, mean % (SD)	29.8 (19.7)
SAPS II score, median [IQR]	38 [29, 46]
SAPS II predicted mortality, mean % (SD)	27.6 (24.5)
APACHE III score, median [IQR]	61 [46, 79]
APACHE III-j predicted mortality, mean % (SD)	28.5 (23.7)
JROD predicted mortality, mean % (SD)	13.5 (16.6)
**Treatments**	
Renal replacement therapy (%)	61 (13.7)
Mechanical ventilation (%)	329 (74.1)
Extracorporeal membrane oxygenation (%)	41 (9.2)
**Outcomes**	
Death at ICU discharge (%)	47 (10.6)
Length of ICU stay, days, median [IQR]	9 [4, 17]
Death at hospital discharge (%)	69 (15.5)
Length of hospital stay, days, median [IQR]	21 [12, 33]

*APACHE* Acute Physiology and Chronic Health Evaluation, *ICU* intensive care unit, *IQR* interquartile range, *JROD* Japan Risk of Death, *SAPS* Simplified Acute Physiology Score, *SD* standard deviation

**Table 2:** Model performance statistics

	APACHE II	SAPS II	APACHE III-j	JROD_**2019**_
AUROC (95% CI)	0.704 (0.634–0.774)	0.696 (0.627–0.765)	0.707 (0.642–0.772)	0.718 (0.654–0.782)
Brier score (95% CI)	0.144 (0.125–0.163)	0.156 (0.125–0.163)	0.155 (0.137–0.174)	0.121 (0.104–0.139)
Hosmer–Lemeshow test, *p* value	< 0.001	< 0.001	< 0.001	< 0.001
Calibration plot				
Slope	0.782	0.472	0.548	0.587
Intercept	−1.124	−1.257	−1.231	−0.452
Standardized mortality ratio (95% CI)	0.521 (0.406–0.660)	0.564 (0.438–0.713)	0.546 (0.424–0.690)	1.151 (0.895–1.456)

*APACHE* Acute Physiology and Chronic Health Evaluation, *AUROC* area under the receiver operating characteristic curve, *CI* confidence interval, *JROD* Japan Risk of Death, *SAPS* Simplified Acute Physiology Score

**Fig. 1:**
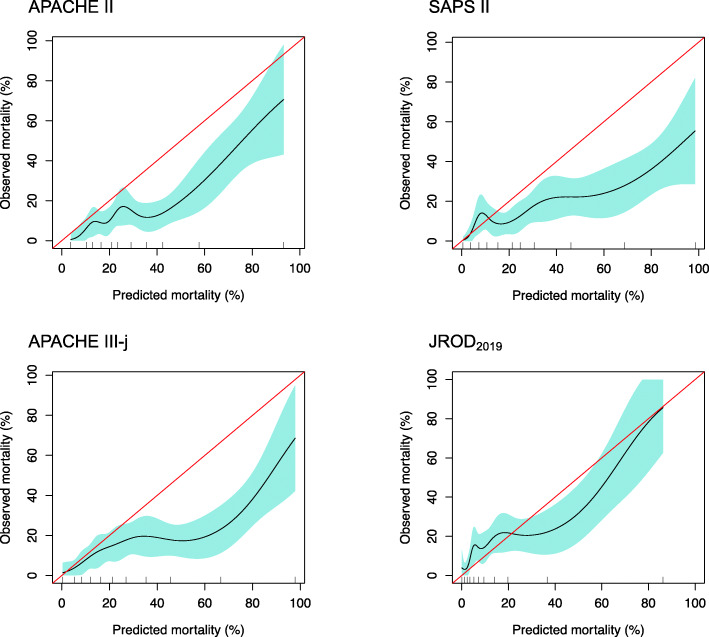
Calibration plots. APACHE, Acute Physiology and Chronic Health Evaluation; JROD, Japan Risk of Death; SAPS, Simplified Acute Physiology Score. Note: Observed mortality is plotted against predicted mortality. The study population was divided according to the predicted mortality into 10 equally sized groups, which are presented as a rug plot along the horizontal axis. A natural spline was drawn for the plots. The shaded area indicates the 95% confidence interval. If the calibration is perfect, the plot aligns with the diagonal line

## Data Availability

The authors’ agreement with the JIPAD project does not allow us to publish the data used for this manuscript or to share it with others.

## Abbreviations

APACHE: Acute Physiology and Chronic Health Evaluation
COVID-19: Coronavirus disease 2019
ICU: Intensive care unit
JIPAD: Japanese Intensive Care Patient Database
JROD: Japan Risk of Death
SAPS: Simplified Acute Physiology Score
